# Preparation and Drug-Loading Properties of Amphoteric Cassava Starch Nanoparticles

**DOI:** 10.3390/nano12040598

**Published:** 2022-02-10

**Authors:** Xinling Xie, Youquan Zhang, Yong Zhu, Yiling Lan

**Affiliations:** Guangxi Key Laboratory of Petrochemical Resource Processing and Process Intensification Technology, School of Chemistry and Chemical Engineering, Guangxi University, Nanning 530004, China; xiexinling@126.com (X.X.); zhuyongty@163.com (Y.Z.); lanyiling1996@163.com (Y.L.)

**Keywords:** cassava starch, W/O microemulsion, amphoteric starch nanoparticles, pH-responsive, drug carrier

## Abstract

Based on the characteristics of charge reversal around the isoelectric point (pI) of amphoteric starch-containing anionic and cationic groups, amphoteric cassava starch nanoparticles (CA-CANPs) are prepared by a W/O microemulsion crosslinking method using (3-chloro-2-hydroxypropyl) trimethyl ammonium chloride as a cationic reagent and POCl_3_ as an anionic reagent, and the effects of preparation conditions on the particle size of the CA-CANPs are studied in detail in the present study. CA-CANPs with a smooth surface and an average diameter of 252 nm are successfully prepared at the following optimised conditions: a crosslinking agent amount of 15 *wt*%, an aqueous starch concentration of 6.0 *wt*%, an oil–water ratio of 10:1, a total surfactant amount of 0.20 g·mL^−1^, and a CHPTAC amount of 4.05 *wt*%. The pH-responsive value of the CA-CANPs can be regulated by adjusting the nitrogen–phosphorus molar ratio in the CA-CANPs. By using CA-CANPs with a pI of 6.89 as drug carriers and the paclitaxel (PTX) as a model drug, the maximum loading rate of 36.14 mg·g^−1^ is achieved, and the loading process is consistent with the Langmuir isotherm adsorption, with the calculated thermodynamic parameters of Δ*H*° = −37.91 kJ·mol^−1^, Δ*S*° = −10.96 J·mol^−1^·K^−1^ and Δ*G*° < 0. By testing the release rate in vitro, it is noted that the release rates of PTX in a neutral environment (37.6% after 96 h) and a slightly acidic environment (58.65% after 96 h) are quite different, suggesting that the CA-CANPs have the possibility of being a targeted controlled-release carrier with pH responsiveness for antitumor drugs.

## 1. Introduction

Starch nanoparticles have a unique size and interface effect in addition to their good biodegradability, biocompatibility, and safety [1,2,3]. They have currently gained widespread use in plastic fillers, implant materials, food additives, food flavor adhesives, biodegradable composite materials, coating adhesives, and emulsion stabilizers [4,5,6,7,8,9].

In recent decades, starch nanoparticles have been used as drug carriers to load ciprofloxacin [10] and diclofenac sodium [11]. The loading capacity and the ability to penetrate biological barriers have been improved, and problems such as the high crystallinity induced by the acid hydrolysis and the high water solubility have been solved. However, these starch nanoparticles had no targeting performance, the drugs were released rapidly during blood transport, and few drug molecules could reach the focus region. Furthermore, glycolysis in tumor cells results in the secretion of large amounts of lactic acid on the plasma membrane, leading to a negative charge on the surface of cancer cells and decrease in pH value in the cancer cells [12]. In particular, the pH values of the lysosome and endosome in cancer cells usually decrease to approximately 5–6 [13]. Drug carriers with amphoteric characteristics can realize the charge reversal function through a pH response at the isoelectric point (PI) [14,15,16]; thus, they can maintain a negative charge in the blood to prolong the blood circulation time of the drug carrier [17]. While near tumor cells, the charge of drug carriers reverses and presents a positive charge, which is easily captured and aggregated by tumor cells [18]. It is expected that the drug carriers with amphoteric characteristics will perform good targeting functions [14].

Some amino acids and proteins have been used to modify starch into amphoteric nanoparticles as targeted drug carriers. For instance, a pH-responsive starch nanoparticle drug carrier with a disulfide bond as the reducing sensitive group was prepared with N, N-diacrylcystamine as a crosslinking agent. After the drug was transported to tumor cells, the disulfide bond in starch nanoparticles was broken, and the drug was released to tumor cells [19]. The corn starch nanoparticle drug carrier for loading insulin was prepared by the ethanol precipitation method and using poly (L-glutamic acid) as the response group [20]. The multilayers of the high-branched cationic and linear cationic starches combined with the whey protein concentrate were constructed for post-loading thocyaninsare [21]. However, as the ratio of carboxyl and amino groups on amino acids or proteins is constant, the isoelectric point cannot be adjusted and the optional amino acids or proteins suitable for the pH variation characteristics of the tumor cell microenvironment are limited. In comparison, amphoteric starches can be easily prepared by introducing cationic groups and anionic groups together. The typical method is to introduce quaternary ammonium cationic groups and phosphate or carboxylic acid anionic groups. The preparation method is simple, and these groups have been successfully used in papermaking, heavy metal and dye wastewater treatment [22,23,24,25,26]. However, there are few reports on the kind of amphoteric starch nanoparticles as targeted drug carriers for loading chemotherapeutic drugs. Moreover, during the preparation of amphoteric starch nanoparticles, by controlling the ratio of anions and cations, the isoelectric points of amphoteric starch nanoparticles can be flexibly adjusted to be suitable for the pH variation characteristics of the tumor cell microenvironment and realize the targeting function with pH-responsive charge reversal.

In the present study, by using cassava starch as a raw material, (3-chloro-2-hydroxypropyl) trimethyl ammonium chloride (CHPTAC) as a cationic reagent, phosphorus oxychloride (POCl_3_) as a crosslinking and anionic reagent, the amphoteric starch nanoparticles (CA-CANPs) were prepared by a W/O microemulsion-crosslinking method. The paclitaxel (PTX) was used as a model drug to investigate the drug loading and release behaviour of CA-CANPs. As expected, the isoelectric point of CA-CANPs was controllable by simply adjusting the ratio of anions and cations; the lower crystallinity of CA-CANPs promotes the drug to be loaded inside nanoparticles, thus improving the drug loading capacity and showing good slow-release performance in a normal body fluid environment. Unstable phospholipid bond hydrolysis under acidic conditions promotes the disintegration of the drug carrier to meet the requirements of faster drug release in a slightly acidic environment.

## 2. Materials and Methods

### 2.1. Materials

Food-grade native cassava starch was obtained from Guangxi Napo Jinyuan Starch Co., Ltd. (Nanning, China). (3-Chloro-2-hydroxypropyl) trimethyl ammonium chloride (CHPTAC) (60 *wt*%), Span80, and Tween60 were purchased from Shanghai McLean Biochemical Technology Co., Ltd. (Shanghai, China). Cyclohexane and sodium hydroxide were supplied by Guangdong Guanghua Technology Co., Ltd. (Guangzhou, China). POCl_3_ was purchased from Sinopharm Chemical Reagents Co., Ltd. (Shanghai, China). Anhydrous ethanol and HCl were obtained from Chengdu Kelong Chemicals Co., Ltd. (Chengdu, China). Paclitaxel (PTX) was supplied by Aladdin Reagents Co., Ltd. (Shanghai, China). All chemicals were analytical grade and used without further purification. Deionized water was used throughout the work.

### 2.2. Preparation of Amphoteric Cassava Starch Nanoparticles

#### 2.2.1. Pretreatment of Cassava Starch by Acid Hydrolysis

A 10 *wt*% cassava starch suspension was prepared from raw cassava starch and 2.2 mol/L HCl solution. The starch suspension was placed into a 40 °C water bath and stirred for 1–72 h; subsequently, the suspension was filtered and washed with deionized water until neutral (pH = 7), and the acid-treated cassava starch granules were obtained by drying in air at 60 °C for 24 h [27].

#### 2.2.2. Preparation of Amphoteric Cassava Starch Nanoparticles (CA-CSNPs)

CA-CSNPs were prepared by a W/O microemulsion-crosslinking method: cyclohexane was used as the oil phase, Span 80 and Tween 60 were used as surfactants, acid-treated cassava starch was used as the raw material, CHPTAC was used as a cationic reagent, and POCl_3_ was used as a crosslinking agent and anionic reagent. First, the pH value of the acid-treated cassava starch suspension (2–10 *wt*%) was adjusted to 11.5~12.0 by using a 1.0 mol/L NaOH water solution, and was gelatinized in a boiling water bath for 40 min to obtain a cassava starch gelatinization solution. Second, the oil phase was prepared by dissolving a 1.0–30.0 g mixture of Span 80 and Tween 60 (Span 80/Tween 60 = 3:7~9:1 (*w*/*w*)) in 100 mL cyclohexane and maintained at 40 °C. Third, at continuous 500 r/min stirring, 5–25 mL of cassava starch gelatinization solution, used as the aqueous phase, was added slowly into the oil phase, and further stirred at 1600 r/min for 1 h to obtain a microemulsion. Subsequently, 3.6–4.2 *wt*% (based on the cassava starch mass) CHPTAC and 5–25 wt% (based on the cassava starch mass) POCl_3_ were added to the microemulsion and reacted at 40 °C for 2 h with stirring at 1600 r/min to obtain the CA-CSNPs microemulsion. Then, the CA-CSNPs microemulsion was demulsified with ethanol and centrifuged at 12,000 r/min with a 5810R centrifuge (Eppendorf, Hamburg, Germany) to obtain the precipitates of the CA-CSNPs. The precipitates were acidified for 0.5 h with 0.2 mol/L HCl and alternately washed with ethanol and ionized water, and the CA-CSNPs were obtained by drying in a vacuum dryer for 5 h at 35 °C.

### 2.3. Characterization

The nitrogen content(*N*%, *w*/*w*) in the CA-CSNPs was determined by an automatic elemental analyzer (Elementar Analysen System, Langenselbold, Germany), and the phosphorus content (*P*%, *w*/*w*) in the CA-CSNPs was determined by ammonium molybdatecolorimetry as described in the literature [28]. The molar ratio of nitrogen to phosphorus (*r_N/P_*) was calculated by Equation (1).

(1) $$r_{N/P}\;=\;\frac{31\;\times \;N\%}{14\;\times \;P\%}$$

where, *r_N/P_* is the molar ratio of nitrogen to phosphorus in the CA-CSNPs; *N*% is the nitrogen content in the CA-CSNPs, *wt*%; *P*% is the phosphorus content in the CA-CSNPs, *wt*%); 14 is the atomic weight of nitrogen; and 31 is the atomic weight of phosphorus.

The morphologies and particle size of native cassava starch, acid-hydrolyzed starch, and CA-CSNPs were characterized by field emission scanning electron microscopy (FESEM, Hitachi SU8220, Tokyo, Japan) at an accelerating voltage of 5 kV. The surface groups on the samples were determined by Spectrum 100 Fourier transform infrared spectroscopy (Perkin Elmer, Waltham, MA, USA). The solid-state ^13^C NMR spectra of the sample after ultrasound treatment for 5 min were obtained by a Bruker AVANCE III 400 HD spectrometer. X-ray powder diffraction (XRD) analysis was performed on a DX-2700A X-ray diffractometer (Rigaku Corporation, Tokyo, Japan) with 40 kV radiation and 30 mA radiation. The average particle size and zeta potential of CA-CSNPs were determined by a Nano-ZS90X laser particle analyzer (Malvern, UK).

### 2.4. Drug Loading and Release on CA-CSNPs

The drug-loading rate of CA-CSNPs was examined using PTX as a model drug. The drug-loading experiment was conducted in the following procedure: 100 mg CA-CSNPs (pI = 6.89) and 2–8 mg PTX were suspended in 50 mL 50–100 *wt*% ethanol aqueous solvent and stirred at 300 r/min at 20–40 °C for 0–420 min. Subsequently, the suspension was centrifuged, and the PTX concentration of the liquid supernatant was determined by a UV–vis spectrophotometer at 230 nm. The drug loading rate (mg·g^−1^) and encapsulation efficiency (%) were calculated by Equations (2) and (3), respectively.

The drug-releasing experiment was conducted in the following procedure: 90 mg drug-loaded CA-CSNPs with a PTX loading rate of 36.14 mg·g^−1^ and 3.20 mg free PTX were added to the dialysis bag respectively, and 10 mL normal saline was added into the dialysis bag. Subsequently, the dialysis bag was placed in a beaker containing 40 mL normal saline and vibrated in a shaker at 37 °C. At an interval of 0–300 min, 40 mL of the solution was removed from the beaker, and the PTX concentration in normal saline was determined by using a UV–vis spectrophotometer at 230 nm. At the same time, 40 mL fresh normal saline was supplied to keep the volume constant. The cumulative release rate of the drug was calculated by Equation (4).

(2) $$q\;=\;\frac{{(C}_{0}{\;-\;C}_{1}{)\;\times \;V}_{0}}{m_{0}}$$

(3) $$y\;=\;\frac{\left(C_{0}\;-\;C_{1}\right)\;\times \;V_{0}}{C_{0}\;\times \;V_{0}}\;\times \;100$$

(4) $$\eta_{d}\;=\;\frac{m_{c}}{m_{1}\;\times \;q_{0}}\;\times \;100$$

where, *q* is the loading rate of CA-CSNPs to PTX, mg·g^−1^; *y* is the encapsulation efficiency of PTX, %; $\eta_{d\;}$ is the cumulative release rate of PTX,%; *C*_0_ is the initial concentration of PTX solution, mg·mL^−1^; *C*_1_ is the residual concentration of PTX solution, mg·mL^−1^; *V*_0_ is the volume of PTX solution, mL; *m*_0_ is the mass of CA-CSNPs, g; *m*_1_ is the mass of CA-CSNPs including loaded PTX, g; *m*_c_ is the cumulative desorption amount of PTX, mg; and $q_{0}$ is the initial load rate of PTX on the CA-CSNPs, mg·g^−1^.

## 3. Results

### 3.1. Pretreatment of Cassava Starch by Acid Hydrolysis

The effects of acid hydrolysis time on the molecular weight of acid-treated starch and the size and yield of CA-CSNPs are shown in Table 1.

Cassava starch has the second-largest molecular weight (only smaller than potato starch) among natural starches, which has a high viscosity of the starch paste. A large number of sticky walls will be found when preparing the W/O microemulsions by using cassava starch, which exhibit a low yield and poor operability. After acid hydrolysis, the molecular weight of cassava starch is reduced, which will greatly alleviate or eliminate wall adhesion. As shown in Table 1, the starch molecular weight, particle size, and yield of CA-CSNPs are significantly affected by the acid hydrolysis time. It was found that cassava starch obtained after 48 h of acid hydrolysis exhibits a distinctly low molecular weight and a small CA-CSNP particle size, which could effectively overcome the wall adhesion phenomenon in the microemulsification process, leading to an increase in the yield to more than 88%, so the product of acid hydrolysis after 48 h is used for further synthesis.

### 3.2. Optimization for The Preparation of CA-CSNPs

**Figure 1:**
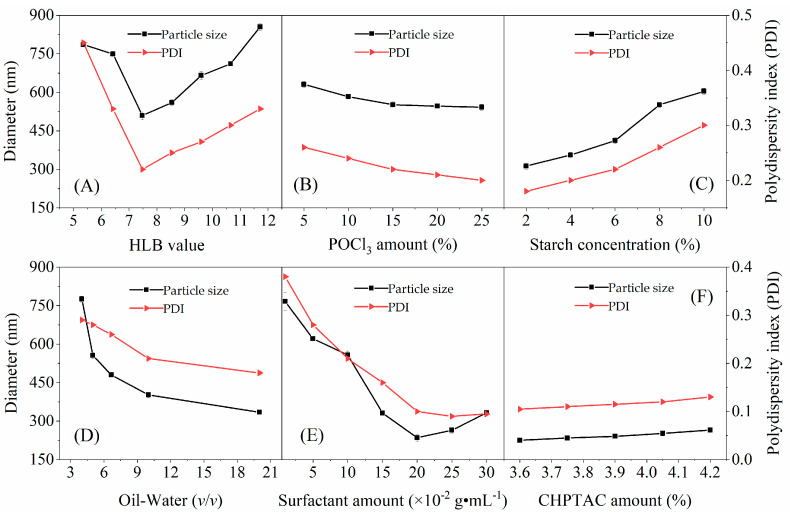
Effects of (**A**) HLB value (at a POCl_3_ amount of 15 *wt*%, an aqueous starch concentration of 6.0 *wt*%, an oil–water ratio of 10:1 (*v/v*), a surfactants total amount of 0.15 g·mL^−1^, and a CHPTAC amount of 3.9 *wt*%); (**B**) POCl_3_ amount (at a HLB value of 7.48, an aqueous starch concentration of 6.0 *wt*%, an oil–water ratio of 10:1 (*v/v*), a surfactants total amount of 0.15 g·mL^−^^1^, and a CHPTAC amount of 3.9 *wt*%); (**C**) starch concentration (at a HLB value of 7.48, a POCl_3_ amount of 15 *wt*%, an oil–water ratio of 10:1 (*v/v*), a surfactants total amount of 0.15 g·mL^−1^, and a CHPTAC amount of 3.9 *wt*%); (**D**) oil–water ratio (at a HLB value of 7.48, a POCl_3_ amount of 15 *wt*%, an aqueous starch concentration of 6.0 *wt*%, a surfactants total amount of 0.15 g·mL^−1^, and a CHPTAC amount of 3.9 *wt*%); (**E**) surfactant amount(at a HLB value of 7.48, a POCl_3_ amount of 15 *wt*%, an aqueous starch concentration of 6.0 *wt*%, an oil–water ratio of 10:1 (*v/v*), and a CHPTAC amount of 3.9 *wt*%); (**F**) CHPTAC amount (at a HLB value of 7.48, a POCl_3_ amount of 15 *wt*%, an aqueous starch concentration of 6.0 *wt*%, an oil–water ratio of 10:1 (*v/v*), and a total surfactants amount of 0.20 g·mL^−1^) on the CA-CSNPs particle size and the polydispersity index (PDI). All reactions were performed at 40 °C for 2 h.

The effects of preparation conditions on the particle size and the polydispersity index (PDI) of CA-CSNPs were studied in detail, and are shown in Figure 1. To make it clearly, we would like to discuss them separately.

As shown in Figure 1A, when the hydrophilic–lipophilic balance (HLB) value of surfactants was lower than seven, the surfactants were highly lipophilic, and the system became turbid or milky, failing to form a microemulsion. When the HLB value was gradually increased to 7.48, the system gradually changed from milky to translucent or transparent and formed a stable microemulsion. As a result, the average diameter of the CA-CSNPs reached the smallest value of 508 nm. When the HLB value exceeded 7.48 and increased to 11.6, a clear and transparent state of the microemulsion was also found; however, the microemulsion system became glassy glue, with high viscosity and poor fluidity, resulting in a larger size. Therefore, the optimal HLB value for preparing the CA-CSNPs was 7.48.

As shown in Figure 1B, when the amount of crosslinking agent (POCl_3_) is increased from 5.0 to 25%, the average diameter of CA-CSNPs gradually decreases from 640 to 550 nm. The observation can be explained as following: with the increase in the amount of crosslinking agent, the crosslinking density of the molecular chains in the CA-CSNPs increase and the molecular arrangement is always compact, resulting in a reduction in the particle size of the CA-CSNPs.

Figure 1C shows that the particle size of CA-CSNPs increases significantly with the increase in starch concentration. This is due to the fact that the higher starch concentration in individual microemulsion micelles would lead to a decrease in the crosslinking density. In this case, the starch molecular chains are loosely arranged, resulting in larger particles. When the starch concentration decreases from 10 to 6%, the average diameter of CA-CSNPs decreases correspondingly from 603 to 396 nm; when the starch concentration decreases from 6 to 2%, the average diameter of CA-CSNPs changes insignificantly from 396 to 326 nm. However, the yield of CA-CSNPs at the starch concentration of 6% was approximately three times higher than that at 2%. Therefore, the selective starch concentration for preparing the CA-CSNPs was 6%.

As shown in Figure 1D, when the oil–water ratio increases from 4 to 10, the average diameter of the CA-CSNPs decreases dramatically from 775 to 402 nm. With the increase in the oil–water ratio, the water phase surface area per unit would obtain more surfactants, thus the interfacial tension would be reduced, making it easy to form smaller droplets. This would eventually lead to a decrease in particle size [29]. However, when the oil–water ratio reaches to 12.5, an insignificant decrease in particle size is observed since the surfactant occupies the optimal proportion at the oil–water interface [30]. Therefore, the selective oil–water ratio for preparing the CA-CSNPs was 10:1.

Figure 1E shows that the particle size of CA-CSNPs decreases when the surfactant amount increases from 0.01 to 0.2 g·mL^−1^. However, an overfull surfactant (>0.2 g·mL^−1^) would form a large number of empty micelles [31], which would trap POCl_3_ molecules and block them to contact the starch molecules effectively, resulting in low crosslinking density and large particle size. Therefore, the optimal surfactant amount for further study was chosen as 0.2 g·mL^−1^.

As shown in Figure 1F, when the CHPTAC amount increases from 3.60 to 4.20%, the particle size shows a slightly increasing trend. Since the cationic and anionic reactions are two competitive reaction processes in a crosslinking reaction, some reactive active sites, i.e., the -OH groups, are occupied by cationic groups, resulting in the decrease in the number of active sites functionalized with the crosslinking agent, and thus the decrease in crosslinking density and a slight increase in particle size.

As for the conditions influencing the PDI of CA-CSNPs, it is shown that the key factors for the formation of the W/O microemulsion, i.e., the ratio of oil to water, HLB value, and the amount of the surfactants, have great influence on the particle size distribution, while the starch concentration, the amount of crosslinking agent, and the amount of CHPTAC have little effect on the particle size distribution. Under the optimized conditions, i.e., the HLB value of 7.48, crosslinking agent amount of 15 *wt*%, aqueous starch concentration of 6.0 *wt*%, oil–water ratio of 10:1, surfactants total amount of 0.20 g·mL^−1^, and CHPTAC amount of 4.05 *wt*%, the obtained CA-CSNPs had a smaller particle size and more uniform particle size distribution, which was characterized in the following and used for the drug-loading properties study.

### 3.3. FTIR and NMR Analysis of CA-CSNPs

In the present work, CHPTAC provides the cationic group, while POCl_3_ provides anionic groups and reacts with hydroxyl groups of the cassava starch molecules in the droplets, working as a crosslinking group between starch molecules [32,33].

**Figure 2:**
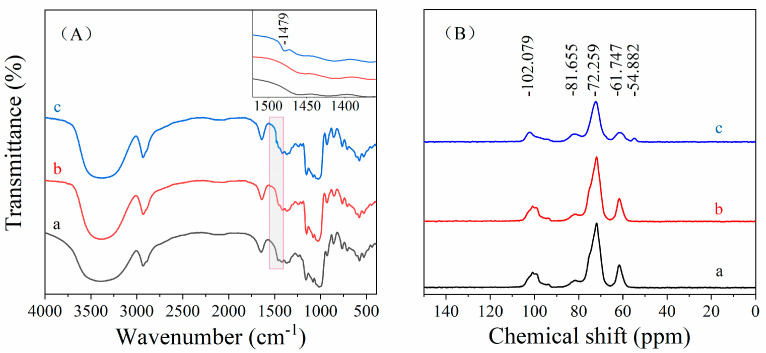
FTIR spectra (**A**) and ^13^C NMR spectra (**B**) of (a) native cassava starch, (b) acid-treated cassava starch, and (c) CA-CSNPs. The inset of (**A**) is a partial enlargement of (a, b, and c).

The FTIR and ^13^C NMR spectra of native cassava starch, acid-treated cassava starch, and CA-CSNPs are shown in Figure 2A and B, respectively.

As shown in Figure 2A, a new peak at 1479 cm^–1^ was found in the spectrum of CA-CSNPs (Figure 2Ac), which is attributed to the stretching vibration of C-H from the quaternary ammonium group [26]. Theoretically, the absorption peaks of P=O and P-O should also appear at 1126 and 1109 cm^−1^ in the FTIR spectrum. However, these peaks are not distinguishable in Figure 2Ac due to the very low content of phosphorus in the samples, which could be shielded by the C-O peaks of cassava starch molecules at 1153, 1078, and 1022 cm^−1^ [34]. Nevertheless, a certain content of phosphorus was detected in CA-CSNPs samples (Table 2), which could be taken as evidence to prove that the crosslinking reaction has taken place between the starch molecule and POCl_3_.

To further confirm the reaction between cassava starch and CHPTAC, the samples were examined using ^13^C NMR spectroscopy, and the results are shown in Figure 2B. The ^13^C NMR spectrum of CA-CSNPs (Figure 2Bc) shows an additional peak at 54.882 ppm, which is attributed to the carbon in the methyl group (-CH_3_) [35]. Overall, these findings indicate that the cationic groups were successfully introduced in the CA-CSNPs, which is consistent with the FTIR results.

### 3.4. Morphology and Crystallinity Analysis

**Figure 3:**
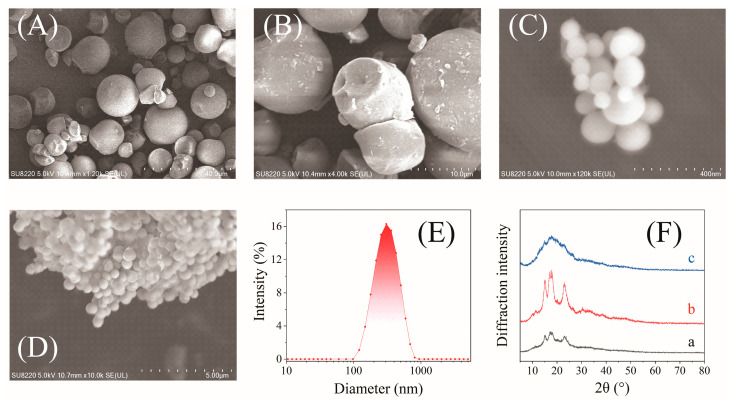
SEM images of (**A**) native cassava starch; (**B**) acid-treated cassava starch; (**C**) CA-CSNPs (×120,000); (**D**) CA-CSNPs (×10.000); and (**E**) the particle size and distribution of CA-CSNPs and the XRD patterns (**F**) of (a) native cassava starch; (b) acid-treated cassava starch; and (c) CA-CSNPs. (The conditions for the preparation of the CA-CSNPs were as follows: a crosslinking agent amount of 15 *wt*%, an aqueous starch concentration of 6.0 *wt*%, an oil–water ratio of 10:1 (*v/v*), a total surfactant amount of 0.20 g·mL^−1^, and a CHPTAC amount of 4.05 *wt*%).

The SEM and XRD analysis results of native cassava starch, acid-treated cassava starch and CA-CSNPs, and the particle size distribution of CA-CSNPs are shown in Figure 3, respectively.

As shown in Figure 3A, native cassava starch particles are round-shaped, while immature cassava starches show irregular particles with particle sizes of 1 to 20 μm. Compared with native cassava starches, acid-treated cassava starches (Figure 3B) show almost the same particle size but with a rougher surface and more broken particles due to fast hydrolysis in the amorphous regions and slow hydrolysis in the crystalline regions [36]. As shown in Figure 3C,D, CA-CSNPs exhibit a smooth surface and uniform particle sizes. Figure 3E shows the results of particle size and distribution of CA-CSNPs measured by a Nano-ZS90X laser particle analyzer (DLS), and the size distribution of CA-CSNPs was relatively concentrated with a polydispersity index (PDI) of 0.12 and an average diameter of 252 nm.

As shown in Figure 3Fa,Fb, strong diffraction peaks were observed in the native cassava starch and the acid-treated cassava starch at 2*θ* = 15, 17, 18, and 23.5°, indicating a typical A-type crystalline structure [36,37]. The crystallinity of the native cassava starch, calculated by the full width at half maximum, was 37.05%, and that of the cassava starch after 48 h of acid hydrolysis was 48.08%, indicating that it is easier for acid hydrolysis in amorphous regions [36]. As shown in Figure 3Fc, the CA-CSNPs retain only weak diffraction peaks at 2*θ* = 15°, and the crystallinity was less than 4.6%, indicating that most of the CA-CSNPs prepared by the W/O microemulsion crosslinking reaction had an amorphous structure.

### 3.5. Thermostability Analysis

**Figure 4:**
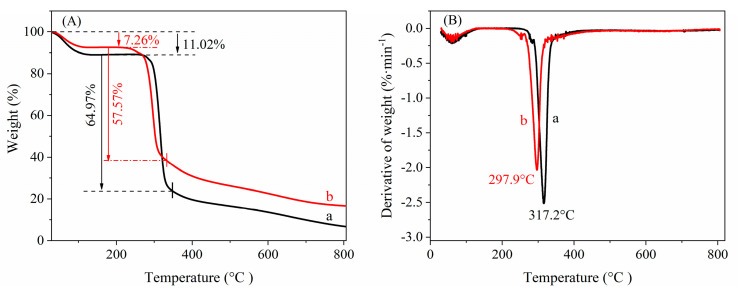
TGA (**A**) and DTA (**B**) of (a) native cassava starch and (b) CA-CSNPs.

Figure 4 shows the TGA and DTA analysis results of native cassava starch and CA-CSNPs.

As shown in Figure 4, the maximum decomposition temperatures of native cassava starch and CA-CSNPs are 317.2 and 297.9 °C, respectively. The results indicated that the thermal stability of CA-CSNPs was only slightly decreased after acid pretreatment, crosslinking, and amphoteric modification of native cassava starch in the W/O microemulsion.

### 3.6. Surface Charge and pH Response Analysis

**Figure 5:**
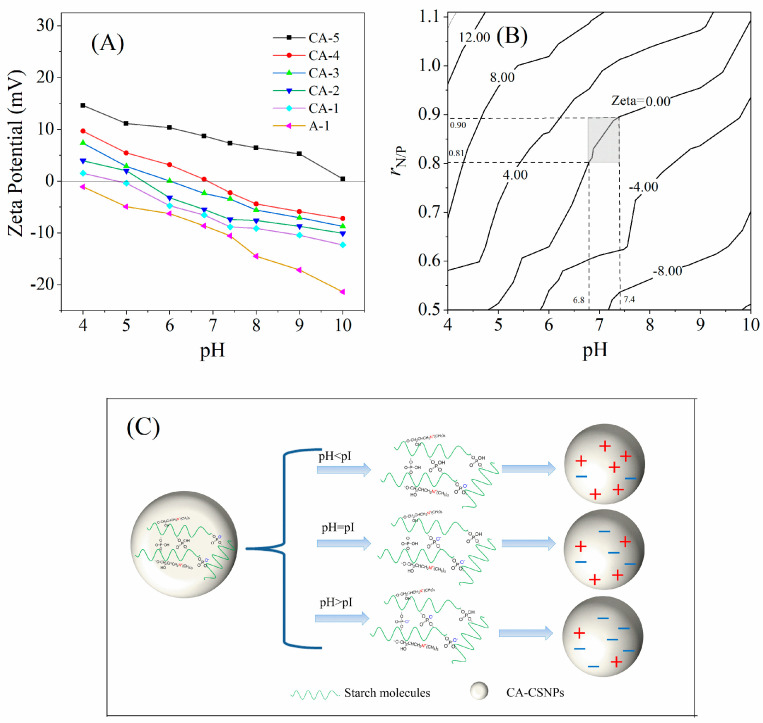
Effects of solution pH and the N/P ratio on the zeta potential (**A**) and isopotential map (**B**) of CA-CSNPs and the charge-reversal mechanism on CA-CSNPs (**C**).

Figure 5A shows that the zeta potential of anionic cassava starch nanoparticles (A-1, *r_N/P_* = 0) is decreasing from −1.12 to −21.37 mV with the increasing of the pH value from 4 to 10, presenting only negative charge characteristics and no isoelectric point. For CA-CSNPs (CA-1~CA-5), the zeta potential is also decreasing with the increase in the pH value from 4 to 10; however, the charge of CA-CSNPs shifts from positive to negative. Thus, the corresponding pH value where the zeta potential value = 0 is the pI of CA-CSNPs. In the left area of the pI line (Zeta = 0) shown in Figure 5B, the zeta potential of CA-CSNPs is positive, and the CA-CSNPs present a positive charge. In the right area of the pI line, the zeta potential of CA-CSNPs is negative, and the CA-CSNPs showed a negative charge. The results indicate that the CA-CSNPs have a charge-reversal function with a pH response, and the pH-responsive value (pI) is increasing with the increase in the molar ratio of nitrogen to phosphorus. As shown in Table 2, when the molar ratio of nitrogen to phosphorus increases from 0.50 to 1.10, the pI of CA-CSNPs increases from 4.79 to 10.32, which indicates that the pI of CA-CSNPs is controllable by adjusting the molar ratio of the cationic and anionic groups on the CA-CSNPs.

The shaded area shown in Figure 5B indicates that the pI of CA-CSNPs could be adjusted to between 6.8 and 7.4 by controlling the *r_N/P_* in the range of 0.81–0.90. Some studies have presented that the pH value of human blood and normal cells is approximately 7.4, while a large amount of glycolysis in tumor cells would secrete a large amount of lactic acid on the plasma membrane of tumor cells, which would not only produce a unique negative charge on the surface of tumor cells but also make the pH of the tumor extracellular microenvironment lower than 6.8, and the pH value of endosomes and lysosomes in tumor cells is approximately 5–6 [12,13]. Therefore, the CA-CSNPs should act as pH-responsive drug carriers to treat tumor cells. As shown in Figure 5C, if the CA-CSNPs are in human blood or near-normal cells, the net charge on the CA-CSNPs surface exhibits a negative charge, which can be conducive to prolonging the blood circulation time of the drug carrier [17]. If the CA-CSNPs is transported to the vicinity of tumor cells, they will conduce the charge-reversal function with a pH response since the pH near the tumor cells is less than 6.8, and the net charge on the surface of CA-CSNPs shows a positive charge, which will promote the absorption capacity of the tumor cells to the drug carrier due to electrostatic attraction [38].

### 3.7. Drug Loading Analysis

Paclitaxel (PTX, C_47_H_51_NO_14_) is a natural diterpenalkaloid with anticancer activity that has been widely used in the treatment of breast cancer, ovarian cancer, some head and neck cancers, and lung cancer [39]. Free PTX in the PTX formulation is easily bound to proteins in the blood and is cleared as a foreign body by macrophages [40]. Biodegradable nanoparticles loaded with PTX as a controlled release formulation for an anticancer drug can achieve various goals, including drug targeting, improving drug availability, and protecting drug bioactivity.

**Figure 6:**
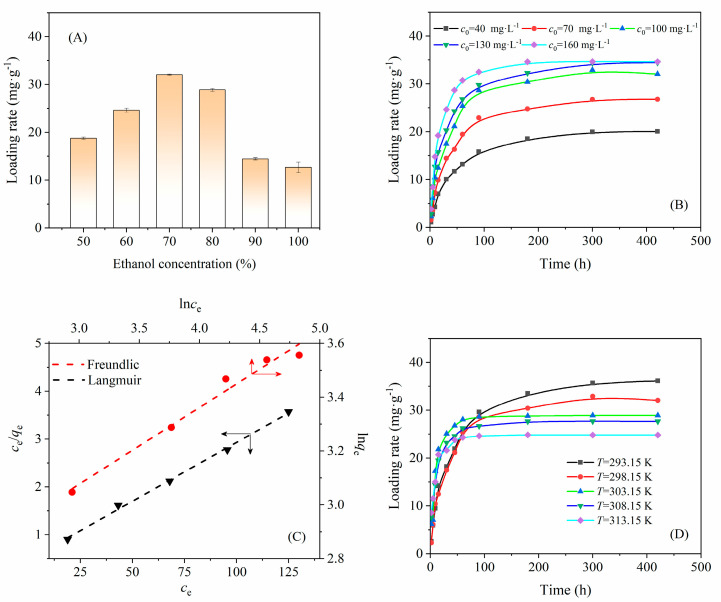
(**A**) Effects of ethanol concentration on the loading rate of CA-CSNPs (*c*_0_ = 100 mg·L^−1^, *T* = 298.15 K); (**B**) effects of the initial concentration of PTX (*c*_0_) on the loading rate (*T* = 298.15 K, concentration of ethanol = 70 *wt*%); (**C**) fitting curves of Langmuir and Freundlich isotherm adsorption equation; (**D**) effects of temperature (*T*) on loading rate(*c*_0_ = 100 mg·L^−1^, ethanol concentration = 70 *wt*%).The CA-CSNPS sample was prepared with a crosslinking agent amount of 15 *wt*%, an aqueous starch concentration of 6.0 *wt*%, an oil–water ratio of 10:1, a total surfactant amount of 0.20 g·mL^−1^, and a CHPTAC amount of 4.05 *wt*%. The obtained CA-CSNPs showed a diameter of 252 nm and an isoelectric point (pI) of 6.89.

The loading rate of the drug by using the adsorption-loading method is mainly affected by the type of media, the initial drug concentration, and the adsorption temperature. The effects of loading conditions on the loading rate of PTX and the fitting results of kinetic adsorption models are shown in Figure 6.

PTX has a low solubility of 30 mg·L^−1^ at 25 °C in water [41] and a high solubility of 39 g·L^−1^ at 25 °C in ethanol [42]. As shown in Figure 6A, PTX are adsorbed better on CA-CSNPs with water and ethanol as the mixed medium, and the maximum loading rate reached 32.07 mg·g^−1^ when the ethanol concentration was approximately 70 *wt*%. The adsorption of ethanol and PTX on CA-CSNPs are competitive adsorption processes; when the concentration of ethanol was less than 70 *wt*%, the solubility of PTX decreased with the increase in water content in an ethanol–water solvent, and some PXT was not dissolved [43], resulting in the initial concentration of PTX being less than 100 mg·L^−1^, and the loading rate at adsorption equilibrium was lower than that of 70 *wt*% ethanol. When the ethanol concentration was higher than 70 *wt*%, although PTX could be completely dissolved in the solvent, the competitive adsorption of ethanol was dominant, leading to a lower loading rate of PTX.

The adsorption of PTX on CA-CSNPs is a reversible process. As shown in Figure 6B and Table S1, when the initial PTX concentration increased from 40 to 160 mg·L^−1^, the time to reach the adsorption equilibrium shortened from 300 min to approximately 150 min, and the loading rate at adsorption equilibrium increased from 21.06 to 35.02 mg·g^−1^; however, the encapsulation efficiency of PTX decreased from 52.65 to 21.89%. From the fitting results of Langmuir (Equation (5)) and Freundlich (Equation (6)) isotherm adsorption equations in Figure 6C and Table S2, the adsorption of PTX on CA-CSNPs was more consistent with the Langmuir isotherm adsorption equation with an *R*^2^ of 0.9974, which indicated that the adsorption of PTX on CA-CSNPs was close to the monolayer adsorption on the ideal surface.

(5) $$\frac{C_{e}}{q_{e}}\;=\;\frac{1}{q_{m}}\frac{1}{K_{L}}\;+\;\frac{1}{q_{m}}C_{e}$$

(6) $$\mathrm{In}q_{e}\;=\;\frac{1}{n}\mathrm{In}C_{e}\;+\;\mathrm{In}K_{f}$$

where, $q_{e}$ is the equilibrium adsorption capacity, mg·g^−1^; *c*_e_ is the equilibrium concentration of paclitaxel, mg·g^−1^; $q_{m}$ is the maximum loading rate, mg·g^−1^; *K*_L_ is the Langmuir adsorption coefficient, L·mg^−1^; *K*_f_ is the Freundlich adsorption coefficient, mg·g^−1^·L^−1/n^·mg^−1/n^; and *n* is the correlation coefficient of the binding energy.

As shown in Figure 6D, when the temperature decreases, the loading rate of PTX on CA-CSNPs at adsorption equilibrium increases accordingly, but it takes a longer time to reach equilibrium. When the temperature decreases from 40 to 20 °C, the time to reach adsorption equilibrium is prolonged from 90 to 400 min, while the loading rate at adsorption equilibrium increases from 24.83 to 36.14 mg·g^−1^. It should be noted that the loading rate of PTX on CA-CSNPs was higher than that on polylactide-hydroxyacetic acid nanoparticles (21.16 mg·g^−1^) [44]. As shown in Table 3, the adsorption equilibrium constant (*K*_L_ or *K*_C_) also decreases with the increasing of temperature. According to Equations (7) and (8), Δ*H*° was calculated to be −37.91 kJ·mol^-1^, Δ*S*° was -10.96 J·mol^−1^·K^−1^, and Δ*G*° < 0, as shown in Table 3, which suggests that the adsorption of PTX on CA-CSNPs was a spontaneous exothermic physical adsorption process and PTX was adsorbed on CA-CSNPs by van der Waals forces and hydrogen bonds between PTX molecules and starch molecules. Therefore, the loading rate would decrease with the increasing of temperature.

(7) $$\ln K_{c}\;=\;-\frac{\Delta H^{{}^{\circ}}}{RT}\;+\;\frac{\Delta S^{{}^{\circ}}}{R}$$

(8) $$\Delta G^{{}^{\circ}}\;=\;\Delta H^{{}^{\circ}}\;-\;T\cdot \Delta S^{{}^{\circ}}$$

where *R* is the universal gas constant 8.314, J·mol^−1^·K^−1^; *T* is the absolute temperature, K; *K*_c_ is the thermodynamic equilibrium constant; Δ*G*° is the changed Gibb’s free energy, J·mol^−1^; Δ*H*° is the changed enthalpy, J·mol^−1^; and Δ*S*° is the changed entropy, J·mol^−1^·K^−1^.

### 3.8. Drug Release Analysis

**Figure 7:**
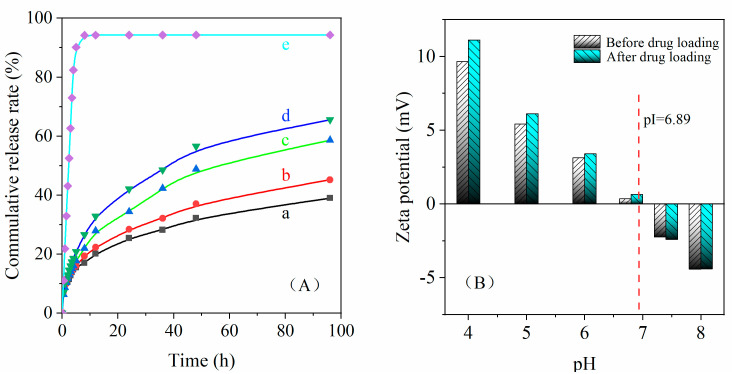
The release rate of PTX (**A**), a. PTX loaded with CA-CSNPs, pH = 7.0; b. PTX loaded with CA-CSNPs, pH = 6.0; c. PTX loaded with CA-CSNPs, pH = 5.0; d. PTX loaded with CA-CSNPs, pH = 4.0; e. Free PTX from dialysis bags, pH = 7.0; and comparing results of zeta potential for CA-CSNPs before and after loading drug (**B**).

The release performance of PTX loaded on CA-CANPs and the comparing results of zeta potential for CA-CSNPs before and after loading drug are shown in Figure 7. As shown in Figure 7Ae, 94.19% of free PTX are released from the dialysis bag in 0.9% normal saline (pH = 7) within 8 h. Compared with free PTX, the release rate of PTX loaded on CA-CSNPs was 17.04% in 8 h and 37.61% within 96 h at the same condition (Figure 7Aa), which indicates that the loaded PTX has a good sustained-release effect in 0.9% normal saline at pH = 7. However, when the pH value of 0.9% saline decreases from 7.0 to 4.0, the release rate of loaded PTX increases from 37.61 to 65.56% in 96 h, which implies that PTX loaded on CA-CSNPs would have a faster release rate when it is transported to the vicinity of tumor cells because of the slightly acidic environment of tumor cells, which is consistent with the micro acid environment tested in vitro.

As can be seen in Table S3, the release kinetics show that PTX release from CA-CSNPs was consistent with the Ritger–Peppas release kinetics (Equation (9)), and the drug release factor (*n*) was 0.32 when the pH value of 0.9% normal saline was 7.0, which suggests that the mechanism of PTX release on CA-CSNPs should be Fick diffusion rather than dissolution desorption [46], and indicates that PTX had already diffused into the carrier interior in the loading process of PTX due to the most amorphous structure of CA-CSNPs. Therefore, once the drug carrier is in the bloodstream, CA-CSNPs will effectively avoid PTX binding with blood protein to protect the physiological activity of PTX. Moreover, it will also reduce the concentration of drug diffused into blood to minimize the damage of PTX to normal cells and enrich more drugs to the target position. However, after the pH value of 0.9% saline decreased to 5.0, the phospholipid crosslink-bond was unstable enough to hydrolyze under acidic conditions, resulting in the fracture of the partial phospholipid crosslink-bond and the disintegration of nanoparticles. In this case, more PTX was delivered, and the drug release factor (*n*) increased above 0.45, indicating that the mechanism of PTX release had changed from Fick type to dissolution desorption. The above results suggest that the CA-CSNPs, as drug carriers of PTX, should have a slow release rate in human blood and a fast release rate in the location of tumor cells, which may meet the application requirements of targeted drug carriers [47].

(9) $$\frac{M_{t}}{M_{\infty }}{\;=\;k}_{H}{\cdot t}^{n}$$

where, $\frac{M_{t}}{M_{\infty }}$ is the cumulative release rate of PTX, %; *t* is the release time, min; *k*_H_ is the release rate constant of the Rigter–Peppas release model, and *n* is the release factor.

### 3.9. Changes in Carrier Isoelectric Point of CA-CSNPs after Loading Drug

As shown in Figure 7B, the zeta potential and isoelectric point of CA-CSNPs (pI = 6.89) before and after loading PTX shows little change due to the lack of ionizable functional groups within the pharmaceutically useful range of PTX [42,48]. However, when the pH value was below the isoelectric point, the zeta potential of CA-CSNPs loaded with PTX was slightly higher than that of CA-CSNPs without PTX, which is due to the positive charge of secondary amino groups in PTX in an acidic environment, and thus would be beneficial to the absorption of drug carriers by tumor cells.

## 4. Conclusions

To investigate the influence of preparation conditions on the particle size of CA-CSNPs in the W/O microemulsion reaction system, an experimental study was performed preparing CA-CSNPs in the W/O microemulsion reaction system with native cassava starch as a raw material, CHPTAC as a cationic agent, and POCl_3_ as an anionic and crosslinking agent. Degrading pretreatment of the cassava starches via acid hydrolysis is necessary to form a stable and uniform W/O microemulsion due to the larger molecular weight and greater branching degree of native cassava starch. By optimizing the reaction conditions, such as the aqueous starch concentration, the oil–water ratio, the amount of crosslinking agent, the amount of CHPTAC, the HLB value, and the amount of emulsifier, CA-CSNPs with a smooth spherical morphology and an average diameter of 252 nm were obtained. The anionic and cationic groups were confirmed to be introduced on the CA-CSNPs by FTIR, NMR and P element detection. The CA-CSNPs also had a charge-reversal function with pH response by the zeta potential test. It is worth noting that the pH responsive value (pI) could be controlled by adjusting the molar ratio of nitrogen to phosphorus. Moreover, the loading and release performance of the CA-CSNPs with a pI of 6.89 were evaluated by using PTX as a model drug. The loading rate of PTX reached 36.14 mg·g^−1^, and the loading process was a spontaneous exothermic physical adsorption process and was consistent with the Langmuir isotherm adsorption equation. In 0.9% normal saline at pH = 7.0, the release rate was 37.61% in 96 h, and the release process of PTX followed the Fick’s desorption mechanism, which implies that PTX diffused into the interior of the CA-CSNPs during the drug-loading process due to the amorphous structure of approximately 95 *wt*%. However, when the pH value of 0.9% saline decreased, the loaded PTX had a faster release rate, i.e., a release rate of 58.65% at pH = 5.0 in 96 h, which is attributed to the hydrolysis of phospholipid crosslink-bonds and the disintegration of nanoparticles under acidic conditions, and the release process changed to a dissolution-desorption mechanism. Hence, the CA-CSNPs could be used as biocompatible, biodegradable control-release carriers for antitumor drugs and have the potential for targeted drug control of tumor cells.

## Figures and Tables

**Table 1:** Effects of acid hydrolysis time on the molecular weight of acid-treated starch and the size and yield of CA-CSNPs.

Acid Hydrolysis Time (h)	Starch Molecular Weight (10^3^ g·mol^−1^)	Diameter (nm)	Yield (%)
0	3708.20	869.5 ± 25.1	57.78
1	2034.48	652.2 ± 10.5	79.64
4	392.51	552.7 ± 6.1	81.00
8	69.31	382.1 ± 6.5	82.88
12	37.28	403.8 ± 9.1	83.12
24	8.71	306.9 ± 11.6	85.12
48	6.60	241.6 ± 3.6	88.60
72	5.32	263.0 ± 10.5	88.58

**Table 2:** Effects of CHPTAC amount on the content of nitrogen and phosphorus, *r*_*N/P*_, and pI of CA-CSNPs.

Samples	CHPTAC Amount (*wt*%)	Nitrogen Content (‰)	Phosphorus Content (‰)	*r_N/P_* (mol·mol^−1^)	pI
A-1	0.0	0.0	4.31 ± 0.02	0.0	-
CA-1	3.60	0.97 ± 0.03	4.27 ± 0.02	0.50	4.79
CA-2	3.75	1.03 ± 0.05	3.96 ± 0.03	0.58	5.38
CA-3	3.90	1.20 ± 0.01	4.20 ± 0.04	0.63	5.95
CA-4	4.05	1.46 ± 0.03	3.81 ± 0.01	0.84	6.89
CA-5	4.20	1.83 ± 0.02	3.68 ± 0.03	1.10	10.32

**Table 3:** Calculated results of thermodynamic parameters from Langmuir isotherm adsorption equation.

*T* (K)	*q*_m_ (mg·g^−1^)	*K*_L_ (×10^−2^ L·mg^−1^)	*K*_c_ (×10^5^)	Δ*G*° (kJ·mol^−1^)	Δ*H*° (kJ·mol^−1^)	Δ*S*° (J·mol^−1^·K^−1^)
293.15	43.99	6.34	14.77	−34.69	−37.91	−10.96
298.15	40.67	5.23	12.18	−34.63		
303.15	39.43	3.82	8.90	−34.58		
308.15	39.84	3.16	7.36	−34.52		
313.15	37.71	2.36	5.50	−34.47		

Note: *K*_c_ is the dimensionless adsorption equilibrium constant [45]; when the ethanol concentration is 70.0 *wt*%, *K*_c_ = 2.33 × 10^7^ *K*_L_.

## Data Availability

Not applicable.

## Supplementary Materials

The following supporting information can be downloaded at https://www.mdpi.com/article/10.3390/nano12040598/s1: Table S1: Effects of the initial concentration of PTX (*c*_0_) on the equilibrium adsorption capacity and encapsulation efficiency; Table S2: The parameter fitting results of the Langmuir and Freundlich isotherm adsorption equations; Table S3: The parameter fitting results of the Ritger–Peppas release kinetic equation.
