# Supplementary material for: Preparation and Drug-Loading Properties of Amphoteric Cassava Starch Nanoparticles
Source: Nanomaterials (Basel). 2022 Feb 10;12(4):598. doi: 10.3390/nano12040598 (PMC8877468; doi:10.3390/nano12040598)
Supplement: Supplementary file 1 [file nanomaterials-12-00598-s001.zip › nanomaterials-1575344-supplementary.pdf]

Supporting information

Preparation and Drug Loading Properties of Amphoteric Cassava Starch Nanoparticles

Xinling Xie, Youquan Zhang*, Yong Zhu, Yiling Lan

Guangxi Key Laboratory of Petrochemical Resource Processing and Process Intensification Technology, School of Chemistry and Chemical Engineering, Guangxi University, Nanning 530004, China

S1. Effects of initial concentration of PTX (c_0) on the equilibrium adsorption capacity and encapsulation efficiency

c_0(mg·L^{-1})	c_e(mg·L^{-1})	q_e(mg·g^{-1})	encapsulation efficiency (%)
40	18.94	21.06	52.65
70	43.22	26.78	38.26
100	67.93	32.07	32.07
130	95.57	34.43	26.48
160	124.98	35.02	21.89

S2. The parameters fitting results of Langmuir and Freundlich isotherm adsorption equations

Model	q_m(mg·g^{-1})	K_L(×10^{-2}L·mg^{-1})	n	K_f	R^2
Langmuir	40.67	5.23			0.9974
Freundlich			3.51	9.22	0.9771

S3. The parameter fitting results of Ritger-Peppas release kinetic equation

pH	K_H	n	R^2
7	8.99	0.32	0.9952
6	9.29	035	0.9991
5	8.61	0.45	0.9906
4	9.53	0.47	0.9928
